# Supplementary material for: Are carbon emissions trading and green financial instruments synergistic? -Comprehensive quantitative research based on content analysis
Source: PLoS One. 2024 Mar 7;19(3):e0298601. doi: 10.1371/journal.pone.0298601 (PMC10919601; doi:10.1371/journal.pone.0298601)
Supplement: S1 Data — (ZIP) [file pone.0298601.s001.zip › Data/地级市Z_F工作报告.zip!/╜¡╬≈/╝¬░▓╩╨/└┤╫╘╣1⁄2╓┌║┼í╛┬φ┐╦╩2╛▌═°í┐_2012.docx]

各位代表：

　　现在，我代表市人民政府向大会作政府工作报告，请予审议，并请各位市政协委员和列席会议的同志提出意见。

　　一、2011年工作回顾

　　2011年是“十二五”规划开局之年。面对极为复杂的国内外经济发展环境的严峻考验，在省委、省政府和市委的坚强领导下，全市上下坚持以科学发展观为指导，紧紧围绕建设开放繁荣秀美幸福新吉安的奋斗目标，深入实施“主攻项目、决战两区，做大总量、加快集聚”重点战略，扎实推进新型工业化、新型城镇化、农业农村现代化“三化”同步互动、三产共生演进，完成了市二届人大七次会议提出的目标任务,实现了“十二五”的良好开局。全市生产总值突破800亿元，达到879亿元，增长12.8%；财政总收入突破100亿元，达到118.3亿元，净增30亿元，增长33.5%，继吉安县之后，新增泰和、安福两县财政收入超10亿元；规模工业主营业务收入突破1500亿元，达到1552.7亿元，增长53.2%；外贸出口总额突破20亿美元，达到20.7亿美元，增长108.2%；旅游总收入突破100亿元，达到124亿元，增长37.8%；金融机构各项存款余额突破1000亿元，达到1052亿元，增长22%。

　　一年来，我们主要做了以下工作：

　　（一）加快产业集聚，结构调整取得新成效。坚持决战“两区”，在做大总量、加快集聚中优化经济结构，提升发展质量，三次产业结构调整为19.3:53.0:27.7，二产比重提高2.5个百分点。工业经济快速增长。全市规模工业增加值330.8亿元，增长19.2%，总量前移一位；利税总额170亿元，增长44%；战略性新兴产业主营业务收入740亿元，增长53%。骨干企业加快壮大，新增主营业务收入过10亿元企业12家，总数达到23家。工业园区加快发展，完成主营业务收入1338亿元，增长44.6%，新增泰和、新干、永丰3个百亿园区，总数达到5个。吉泰走廊完成工业主营业务收入880.6亿元，井开区完成226.2亿元，分别占全市总量的56.7%和14.6%。农业经济稳步发展。全面落实强农惠农政策，农业生产能力进一步增强，新增和改善灌溉面积37万亩，改造中低产田14.3万亩。农业生产获得全面丰收，粮食总产385.4万吨，连续8年增产，生猪、家禽、蔬菜、水果、水产等主要农产品产量均创历史最高水平。特色产业快速发展，新增井冈蜜柚2.8万亩、高产油茶10.2万亩、花卉苗木4万亩，57个农产品获中国驰名商标、江西省名牌产品及著名商标。农业产业化进程加快，新增市级以上农业龙头企业23家，达到246家，新增农民专业合作社309个，达到1535个。现代服务业日益繁荣。旅游业实现新突破，庐陵文化生态园、井冈山杜鹃山评为江西省十大新旅游景区；全市接待国内外游客1830万人次，增长29.4%，井冈山门票收入突破2亿元，增长97%，井冈山机场旅客吞吐量30.1万人次，增长65.4%。商贸市场建设扎实推进，农村流通服务网络不断完善，城乡消费活跃，全市社会消费品零售总额228.5亿元，增长17.7%。

　　（二）坚持城乡统筹，城镇化迈出新步伐。围绕“文化庐陵、山水吉安”的城市定位，大力推进城镇建设和美丽乡村建设。全市实施重点城建项目258个，完成投资125.2亿元，城镇化率提升2.03个百分点，达到39.62%。中心城区建设步伐加快。建成区面积达到59.6平方公里，新增2.9平方公里。市文化艺术中心被评为江西省首届十佳建筑；市民广场、吉安桐坪机场、城南新区和滨江新区路网、城市主干道街景改造等工程竣工；93个基础性、功能性、生态性的城建项目扎实推进，完成投资41.3亿元；森林城市创建工作有序开展，绿化、美化、亮化工程有效实施，新增公园绿地面积115.4万平方米，中心城区功能不断完善，品位不断提升。县市城区建设进一步提速。园林绿化、街景改造、商贸市场、污水垃圾处理等工程全面实施，新增园林绿地面积410.8万平方米，新建污水管网189.5公里，遂川县创建省级园林县城，全市省级园林县城达到9个。城乡同治成效明显。31个示范镇、重点镇加快建设，6个乡镇评为国家级生态乡镇；913个新农村建设任务全面完成，打造了一批具有庐陵风格的美丽乡村，5个村获全省人居环境范例奖，井冈山昌蒲村评为中国最有魅力乡村，吉水县低坪村评为全国文明村镇。

　　（三）实施项目带动，投资规模实现新扩张。全市固定资产投资739.2亿元，增长30.2%。争资争项成效显著。争取国家和省支持的各类项目827个、中央和省建设资金15.3亿元。项目建设顺利推进。列入全市调度的420个重点项目完成投资405亿元，增长26.6%。国省道改建及路面重建、农村公路建设、青东公路改造一期工程、西气东输二线工程、10个输变电工程等项目顺利完工；峡江水利枢纽、石虎塘航电枢纽、衡茶吉铁路、吉莲高速、抚吉高速、井睦高速等重点工程快速推进；105国道中心城区改道、永和连心大桥、井开区深圳大道跨铁路大桥等项目开工建设。工业投资势头强劲。全年完成工业投资535.7亿元，增长38.8%，占全市投资总量的72.5%。三菱超纤维、博锐泰光电科技、长虹电线电缆、永丰南方水泥等一批重大产业项目竣工投产，杰彼新能源、中信华电子、宜华木业等一批超10亿元项目进展顺利。

　　（四）推进改革开放，体制机制创新取得新进展。各项改革深入推进。非工口七个系统144户国有企业改革全面完成，安置职工2.7万人。医药卫生改革扎实有效，全市234所乡镇卫生院实现国家基本药物制度全覆盖。林业产权配套改革不断深化。文化体制改革稳步推进，市采茶剧团与市歌舞团顺利合并，组建吉安市采茶歌舞剧院。事业单位人事和工资制度改革有序实施，岗位设置圆满完成，绩效工资改革进展顺利。金融创新成效明显。吉安农村商业银行、赣州银行、吉州珠江村镇银行等一批金融机构顺利开业，小额贷款公司实现县域全覆盖。年末金融机构各项贷款余额450亿元，增长20.9%。对外开放大步迈进。全年实际利用内资248.4亿元，增长33.6%，实际利用外资5.03亿美元，增长14.2%。在力抓工业招商的同时，积极拓展城市建设、现代农业、现代服务业等领域招商，引进亿元以上工业项目156个，总投资770亿元，引进重大城市建设项目、农业产业项目、现代服务业项目37个，总投资193亿元。对外贸易大幅增长，进出口总额22.96亿美元，增长103.3%。

　　（五）强化民生保障，群众生活得到新改善。全市城镇居民人均可支配收入17692元，增长13.8%，农民人均纯收入6308元，增长13.3%。民生工程深入实施。省政府下达的66项民生实事全面完成。就业渠道不断扩大，购买公益性岗位6065个，发放小额担保贷款5.8亿元，新增城镇就业8.7万人，转移农村劳动力17万人。社会保障体系日益完善，农村新型养老保险、城镇居民养老保险试点工作扎实推进，参加五项社会保险人数达443万人，增长53%。社会救助水平不断提高，城乡低保对象月人均补差额分别增长12.7%和23.4%。保障性安居工程加快推进，建成经济适用房、廉租房、公租房6823套，改造农村危房10100套。农村饮水安全工程成效明显，解决了24.89万人的饮水安全问题。移民扶贫开发稳步推进，完成移民搬迁1.45万人。峡江水利枢纽工程110个移民安置点有84个启动建设。生态建养全面推进，“一大四小”工程建设完成造林61.4万亩。节能减排有效推进，中心城区环境空气质量保持在国家二级标准以上，主要河流水质保持在国家三类标准以上，饮用水源达标率100%。

　　（六）注重协调发展，社会事业有新进步。科教兴市战略深入推进。义务教育稳步提升，高考成绩创撤地设市以来最好水平；开发国家和省重点新产品44项，新设2个省级民营科技园，获得4项省级科技奖。文化、体育、卫生事业加快发展。建成乡镇综合文化站105个、农家书屋750家，永丰、永新、吉安三县被命名为“中国民间文化艺术之乡”，新干剪纸列入第三批国家级非物质文化遗产名录；全民健身活动深入开展，成功举办“七城会”男子篮球（U16）赛；新型农村合作医疗参合农民358万人，参合率97.6%；“光明·微笑”工程和儿童“两病”免费救治工作积极实施，尿毒症患者免费透析工作全面启动。人口与计划生育、食品药品监管、安全生产、社会治安综合治理等工作有效加强。人口自然增长率控制在7.51‰；连续8年未发生等级以上食品药品安全事故；安全生产形势平稳，信访工作和公安“清网行动”成效显著，社会保持稳定。国防动员、民兵预备役、征兵、人民防空等工作不断推进，我市包括井冈山市被评为全国双拥模范城。审计、监察、统计、物价、民族宗教、对台事务、外事侨务、检验检疫、海关、质监等工作不断加强，精神文明、广播电视、邮政通信、档案、地方志、供销、气象、水文、公积金等工作不断发展，妇女、儿童、老龄、残疾人事业不断进步。

　　过去一年，政府自身建设得到进一步加强。依法行政扎实推进。制定出台《关于加强法治政府建设的实施意见》，将依法行政纳入考评体系。开展行政强制规定清理，废止和修改规范性文件26个。自觉接受市人大及其常委会的法律监督、工作监督和市政协民主监督，办理省、市人大代表建议和政协提案357件，办结率达100%。行政效能得到提升。深入开展了“发展提升年”及“思想大解放、作风大转变、服务大提升”主题教育活动。进一步完善政府重大事项决策程序和新闻发布制度，稳步推进政府信息公开。加强了行政服务中心和公共资源交易中心建设，网上审批和电子监察系统运行良好。群众诉求渠道进一步畅通。开通行风热线和网络在线访谈，完善市长直通电话、市长信箱、市长手机受理办理工作机制，全年共受理办复群众来信来电4.3万件次。惩治和预防腐败体系建设不断加强，党风廉政建设责任制有效落实，风险岗位廉能管理成效明显，政务环境不断改善。

　　各位代表，过去一年的成绩来之不易，是省委、省政府和市委科学决策、正确领导的结果，是市人大、市政协有效监督和大力支持的结果，是全市上下团结拼搏、共同奋斗的结果。在此，我代表市人民政府，向奋斗在各条战线的全市广大干部群众，向各位人大代表、政协委员，向历任老领导、离退休老同志，向各民主党派、工商联、无党派和社会各界人士、各人民团体，向驻吉人民解放军、武警官兵、公安干警，向中央和省驻吉单位，向所有关心、支持和参与吉安建设的同志们、朋友们，表示崇高的敬意和衷心的感谢！

　　在看到成绩的同时,我们也清醒地认识到，经济社会发展中还面临不少问题和困难。欠发达的基本市情没有根本改变，经济总量偏小仍然是我们面临的主要矛盾和问题；资源环境约束日益凸显，土地、资金、劳动力等生产要素将是长期制约发展的突出问题，农村耕地保护和农民建房管理面临严峻挑战；影响社会和谐稳定的因素仍然较多，创新社会管理面临许多新课题；经济发展环境还有不尽如人意的地方，政府推进科学发展、依法行政的水平有待进一步提高。对这些困难和问题，我们将采取更加有力措施，认真加以解决。

　　二、2012年的工作任务

　　2012年是实施“十二五”规划承上启下的重要一年，是贯彻市第三次党代会战略部署的关键之年。今年的宏观经济形势更为复杂，保持经济平稳较快发展面临诸多挑战和考验。世界经济复苏的不确定性因素较多，加上贸易保护主义倾向更加突出，我们开拓国际市场难度增大；国内经济发展中不平衡、不协调的矛盾和问题仍然突出，节能减排形势严峻，经济增长下行和物价水平上行的压力仍然较大；地区间的竞争更趋激烈，生产要素的保障难度加大。但总体看，我国经济发展长期向好的基本面不会改变，我市加快发展的有利条件多于不利因素。随着经济全球化深入发展，国际资本和产业加快转移，我们仍处于重要战略机遇期；在发达国家经济复苏艰难的情况下，国际资本将会更多地转向发展中国家寻求投资机会，我们利用外资、引进先进技术和管理经验面临更多机遇；国家坚持稳中求进的工作总基调，实施积极的财政政策和稳健的货币政策，继续加大对“三农”、保障性住房、社会事业等领域的投入，继续支持欠发达地区、战略性新兴产业、重大基础设施、企业技术改造等，为我们争取政策、项目、资金提供了有利条件；随着我市一批重大基础设施和产业项目建成投产，为经济社会发展增添了强大后劲；特别是市第三次党代会的胜利召开，以及通过一系列主题教育活动，全市上下精诚团结、奋发作为的氛围更浓，抓项目、谋发展、促和谐的干劲更足，为今后发展蓄积了强大精神动力。只要我们坚定信心、抢抓机遇，趋利避害、扎实苦干，就一定能推动经济社会发展再上新水平，迈出建设开放繁荣秀美幸福新吉安的坚实步伐。

　　2012年政府工作的总体要求是：全面贯彻省第十三次、市第三次党代会精神，深入贯彻落实科学发展观，积极策应鄱阳湖生态经济区建设，紧紧围绕建设开放繁荣秀美幸福新吉安的奋斗目标，坚持“主攻项目、决战两区，做大总量、加快集聚”，坚持工业和服务业双轮驱动，坚持增加投入和扩大内需并重，深入推进井开区、吉泰走廊建设和县域经济发展，促进三化同步互动、三产共生演进，促进经济结构调整，深化改革开放，优化生态环境，保障改善民生，确保经济平稳较快发展和社会和谐稳定，以优异成绩迎接党的十八大胜利召开。

　　按照上述要求，坚持主要指标增幅略高于全国、全省平均水平，今年全市经济社会发展的主要预期目标是：生产总值增长12%，财政总收入增长20%，地方财政收入增长18%，固定资产投资增长25%，规模以上工业增加值增长18%，社会消费品零售总额增长18%，实际引进市外资金增长18%，实际利用外资增长10%，外贸出口增长10%，城镇居民人均可支配收入和农民人均纯收入均增长12%，城镇化率提高2个百分点，居民消费价格总水平涨幅控制在4%左右，人口自然增长率控制在7.5‰以内，单位生产总值能耗下降3%，化学需氧量、氨氮排放量、二氧化硫排放量、氮氧化物排放量分别下降1%。

　　围绕上述目标，重点抓好以下八个方面的工作。

　　（一）推进吉泰走廊建设，打造加快发展的核心增长极。抓住吉泰走廊纳入全省区域发展格局的机遇，集中优势资源、优势力量，加快挺起吉泰走廊脊梁，引领带动全市发展。

　　加快昂起井开区龙头。围绕决战千亿园区，着力提升产业聚集的基础条件，重点实施园区道路、陆地港、物流中心、电子市场、梨山公园二期、污水处理厂等十大配套工程。抓紧建设出口加工区，10月份通过国家验收并封关运行。推进产业项目建设，抓好施立科新材料、创成科技等29个新建续建项目，力争航盛电子工业园一期工程竣工投产，通讯终端产业城建成标准厂房12万平方米。抓好电子信息、生物医药、先进制造等主导产业招商，引进重大工业项目20个以上。推进井开区与深圳南山区、杭州萧山区的对接合作，加快建设南山工业示范区。扶优扶强骨干企业，新增主营业务收入超50亿元企业1家，超10亿元企业9家。力争井开区规模工业主营业务收入达300亿元。

　　加快建设电子信息产业基地。围绕打造在全国有影响力的电子信息产业基地，以井开区为龙头，引领带动其他园区突出特色、错位发展，构建优势互补、配套协作、结构合理的电子信息产业体系。在井开区重点发展通讯终端、车载电子产业，支持其余三县两区依托产业基础，在计算机配件及整机、通讯设备和电子元器件、LED照明、液晶显示器、电子电镀集控等方面定位主攻，形成优势产业板块。加大重大项目招商引进、协调推进力度，着力抓好惠乐光电、泰屹电子等40个亿元以上在建项目，年内竣工投产20个以上；引进亿元以上项目26个以上。力争吉泰走廊电子信息产业主营业务收入260亿元，井开区120亿元。

　　加快构建吉泰城市群。立足“大吉安”城市格局，以中心城区为核心，以吉水、泰和为两翼，以周边镇为卫星镇，推进吉泰城市群建设。坚持规划引领，高起点编制吉泰走廊建设总体规划以及城镇体系、产业发展、基础设施、土地利用、空间利用、社会事业等8个专项规划。加快完善中心城区承载功能，提升吉水、泰和城关镇发展水平，力争城镇化率提高2.5个百分点。着力打造吉泰走廊卫星镇，按照“九个一”要求，先行抓好吉水县黄桥、金滩镇，青原区天玉、新圩镇，吉州区兴桥、长塘镇，吉安县永和、凤凰镇，泰和县沿溪、螺溪镇等10个卫星镇的基础设施建设，发挥示范作用。大力推进走廊区域公交、电力、社会保障等基础设施和公共服务一体化，年内开通中心城区、各县城到走廊区域内乡镇、工业园区的公交线路；抓好7个220千伏等输变电工程及相关电网建设；加强社会保障和就业服务平台建设，构建市、县、乡劳动力市场三级联动服务体系。

　　（二）坚持工业主导，打造进位赶超的强力引擎。围绕“十二五”决战主营业务收入4000亿元，打造“百亿园区、千亿产业”，推进工业上规模、上能级、上水平。

　　壮大提升支柱产业。依托产业基础和资源优势，加快冶金建材、医药化工、机械制造、食品加工等传统优势产业提升改造，壮大规模、延长链条，力争“6+1”支柱产业主营业务收入达到1500亿元。重点抓好华邦铜业20万吨高档铜制品、祥丰化工离子膜烧碱、唯冠油压扩建、博世顿液压机械、三一重工机械整机与零部件生产、金冠食品加工等80个重大项目建设，竣工投产50个以上。支持龙头企业通过产能提升、技术改造、裂变扩张等方式做强做大，力争主营业务收入过亿元企业达到400家以上，过10亿元企业30家。

　　加快发展战略性新兴产业。以高新、高端、高科为方向，推进战略性新兴产业项目集群、产业延伸，重点加快新能源及节能技术、新医药及微生物农药、新材料等领域产业集聚。完善用地、融资、财税扶持等政策措施，着力抓好杰彼新能源太阳能电池及跟踪发电系统、金一泰电动车、腾胜科技太阳能电池板、华因康基因科技产业园、量科照明节能产品等60个战略性新兴产业项目建设，竣工投产40个以上，力争战略性新兴产业主营业务收入达到900亿元。

　　推动工业园区发展升级。大力推进标准厂房、污水处理、供电网络、供水供气等设施建设，提升产业承接保障能力。推进科技入园，支持企业技术创新，抓好10个省级创新型试点企业建设，开发省级新产品20项以上。全面推进生态园区建设，抓好企业节能减排，注重园区绿化、美化和环境保护，新增2个省级生态工业园区。促进园区节约集约用地，完善土地管理机制，把住准入门槛，提高投资强度，对未按时开工或实施不到位的入园项目，实行土地回购。支持用地不足的园区争取扩区调整政策，拓展发展空间。力争全市工业园区主营业务收入达1600亿元，新增4个百亿园区，总数达到9个。

　　（三）大力发展现代服务业，培育新的经济增长点。坚持主导产业抓集聚、重点产业抓规模、新兴产业抓培育，打造赣中现代服务业重要集聚区，力争服务业增加值突破300亿元。

　　提升发展商贸流通业。完成市县两级商贸市场体系规划，推进市场建设。繁荣中心城区商贸业，实现新世界商城建成开业，天虹商城、华润商城主体工程完工；加快“十大专业市场”建设，基本建成吉安农副产品物流中心、城北和城东汽车综合市场、城南生产资料主体市场、青原区电子数码产品专业市场主体工程，开工建设吉泰走廊高档家装家俬市场、吉福路小商品批发市场，招商建设吉安文化教育图书市场、木材交易市场。抓好县市区专业市场和流通网络建设，基本形成覆盖县、乡、村三级的商贸市场体系。

　　大力发展现代物流业。加快推进“一中心、一平台、六园区”建设。抓紧建设井开区现代物流中心，完善仓储、联运、配送等功能，引进投资主体建设第四方物流平台，实行横向联网、信息共享。完成吉安县、泰和县物流园区一期工程，开工建设青原区、峡江县、吉水县、新干县等物流园。支持引导交通运输和商贸企业拓展物流功能，加速形成相互连接、集聚发展的物流业体系。

　　加快发展旅游业。实施以井冈山红色旅游为龙头，青原山、武功山互动并进的“三山”战略。大力发展井冈山红色旅游，拓展红色经典培训和会展经济，深度开发绿色生态休闲旅游产品，推进井冈山旅游上市，支持申报世界自然和文化遗产。高起点完成青原山景区开发建设规划，妥善解决制约景区发展的有关问题，推进景区重点项目建设。推进武功山综合开发，抓好景区索道、游客服务中心等重点项目建设，完成嵘源温泉度假村二期工程并启动三期建设。拓展中心城区旅游，组建中心城区旅游公司，建设游客服务中心，完善庐陵文化生态园游乐服务设施，建设后河“金腰带”、螺湖湾、庐陵美食城、仓口庐陵特色街。加快发展古村文化游、绿色生态游和美丽乡村游。力争全市旅游接待总人次突破2000万，旅游总收入突破150亿元。

　　积极发展文化产业。发展文化创意产业，完成天腾科技创意文化产业园一期工程，开工建设奥飞动漫产业园。发展文化经营业，提高市文化艺术中心、市体育馆经营水平；建立市采茶歌舞剧院与艺术学校联动机制，提升演艺实力。发展新闻传媒业，加快建设井冈山报业大厦、市文广传媒中心。发展文化产品制造业，争取设立建设国家蓝光技术研发和光盘生产基地，引进一批音像制品生产企业；充分挖掘红色文化、庐陵文化底蕴，开发生产特色文化产品。

　　统筹发展金融业和新兴服务业。进一步优化金融生态环境，大力开展金融招商，争取新设交通银行吉安支行。加强银企对接，鼓励和支持各类金融机构开展业务创新，扩大信贷规模，全年新增贷款增长20%。进一步发挥担保公司作用，扩大融资担保规模。发挥政府六大投融资平台作用，通过债券、基金等方式积极融资。积极利用海外银行和国际金融组织的信贷资金。大力推进企业上市，做好上市企业辅导帮扶和上市报批工作，力争取得实质进展。统筹发展服务外包、电子商务、中介服务、社区服务、总部经济等服务业，实现各种业态良性互动、共同发展。

　　（四）着力决战城区，加快新型城镇化进程。以“四城同创”为抓手，注重融入文化内涵和绿色生态特色，强化规划管控，提升城镇建设管理水平，加快打造大气吉安、雅气吉安、秀气吉安。

　　加快中心城区建设。实施93个重点城建项目，决战城南新区建设，建成市行政中心等八大公共建筑，开工建设商业慢行系统、金融街区、五星级酒店、体育中心、高职学院、城南医院、城南住宅小区，带动新区集聚成形；抓好赣江西堤综合改造，重点实施一期工程，开工建设路堤结合改造、跃进路南延工程。加快后河“金腰带”建设，完成二期主体工程。积极推进河东滨江新区开发，完成滨江大型超市零售商城主体工程，开工建设滨江生态健身公园。推进城北新区建设，加快商贸物流开发，实施螺湖湾沿河景观带建设。支持敦厚片区向北拓展，加速融入中心城区。继续实施老城区“五位一体”提升改造工程，重点抓好小街小巷及74个老住宅小区的综合改造。实施中心城区人口五年倍增计划，全面放宽城镇户籍限制，吸纳更多农业人口和各类人才在城区就业落户。

　　加强县市城区和小城镇建设。坚持交通和绿化先行，以完善功能、提升管理为重点，拓展城镇框架，做大城镇规模。健全规划体系，全面完成县市城区总体规划修编，配套完善各类专项规划、详细规划，近期建设地段控制性详规覆盖率达到100%。抓好县市城区项目建设，高标准建好一条出城主干道、一条城中景观带、一个商业集聚区、一座广场公园。实施城市美化亮化、园林绿化、老城区改造提升、城区管护工程，力争全部县市城区达到省级园林城市标准。抓好省市示范镇、民族乡、重点镇及各县市区中心镇建设，完善基础设施，提升公共服务能力。

　　推进“四城同创”。以创建国家森林城市、国家生态园林城市、国家卫生城市和省级文明城市为抓手，坚持以人为本、建管并重，建设秀美吉安。深入实施造林绿化“一大四小”工程，稳步提高森林覆盖率和城镇绿化率，打造城市“绿肺”。抓好单位院内空地、居民小区空地、城市边角地、城郊荒坡地的绿化，以及较宽人行道、大型商场、单位广场、商贸区等区域的退硬还绿、立体绿化、垂直绿化，做到见缝插绿、拆墙透绿、能绿则绿；抓好农村村庄、农家庭院和通道绿化工作，提升乡村绿化水平。力争4月底前，市中心城区达到国家森林城市创建标准，其他县市基本达标。大力推行城镇精细化管理，重点强化规划实施、环境卫生、市容市貌、小区物业、道路交通管理措施。维护规划的严肃性，在土地供应、项目审批、建筑设计等环节严格落实规划要求，严厉查处违章建筑、违法用地；对两年以上不投资或不开工的城镇闲置土地实行清理。建立健全市容环境卫生监督考核长效机制。实施城乡洁净工程，集中开展卫生大整治，突出抓好城乡结合部和停车场、餐饮店、夜市、农贸市场等周边的环境卫生治理，消灭卫生盲区和死角；整治市容市貌，推进街景综合改造，完善环卫基础设施建设，加强建筑工地和建筑余土运输管理。注重物业市场的培育与监管，着力解决小区安全防护和老旧小区无人管理等突出问题。

　　（五）推进美丽乡村建设，加快农业农村现代化。坚持城乡统筹、城乡同治，加大“三农”工作力度，促进农业增效、农民增收、农村发展，推动农业大市向农业强市转变。

　　积极发展现代农业。严格耕地保护，确保粮食播面稳定在960万亩以上，粮食总产稳中有升。积极引导农村土地向专业大户流转，促进农业规模化、集约化发展。加大奖补扶持力度，培育壮大高产油茶、井冈蜜柚、花卉苗木、绿色蔬菜等特色产业，构建“一县一业”、“一村一品”发展格局，年内新增高产油茶11万亩，井冈蜜柚、花卉苗木、蔬菜种植面积各5万亩。抓好井冈山国家农业科技园和6个现代农业产业园建设，推动设施农业、休闲观光农业发展，打造一批现代农业示范县和示范村镇。加快建设各种用材林和商品林基地，积极发展木材精深加工业和林下经济。促进龙头企业发展壮大，力争全市产值超亿元龙头企业达到35家。推进农民专业合作社规范化建设，新增农民专业合作社200个。扶持农产品品牌，争创20个以上绿色农产品，30个以上无公害认证标志。

　　提高农业综合生产能力。加快水利改革发展，着力抓好小型农田水利和灌区续建配套等五大工程建设，重点推进新开工的312座病险水库除险加固、5个中小河流治理以及126个续建工程，全面完成水利设施三年大会战任务。加快高标准农田建设，积极争取并整合涉农项目资金，新建高标准农田16万亩。推进农业科技创新，加大农业专业人才培养和引进力度，加快农技推广步伐，完善农村防灾减灾、农业气象、农产品质量安全监管等服务体系。

　　推进美丽乡村建设。按照“生态美、村容美、庭院美、生活美、乡风美”的要求，制定和完善美丽乡村建设规划，全面完成30户以上自然村村庄建设规划。重点在省市示范镇、吉泰走廊重点镇、各县市区中心镇和交通干道沿线乡镇进行选点布局，启动850个新农村点建设，大力实施镇村联动、村落连片和大村整体推进，打造美丽乡村样板。加强村庄规划建设管理，抓好农村社区建设和空心村整治。加大农民建房管控力度，严格落实“八个禁止”，强化准入管理，加强对农民建房条件、房屋设计、施工队伍和建筑工匠选择的审查，实行县、乡、村“三位一体”的建房审批监管制度。

　　抓好新一轮扶贫开发。围绕“三不愁、三保障、五突破”总体目标，大力实施专项扶贫、行业扶贫、社会扶贫，建立帮扶制度，改善贫困地区生产生活条件，增强自我发展能力。积极争取中央苏区振兴政策。抓好罗霄山脉集中连片特殊困难地区扶贫开发工作。完成片区扶贫开发总体规划，启动一批扶贫攻坚项目。实施661个贫困村整村推进扶贫开发。加大深山区、地质灾害区搬迁移民扶贫力度，完成1万人以上搬迁安置。做好峡江水利枢纽库区移民安置工作，年底前基本完成110个移民新村建设任务。

　　（六）深化改革开放，增强发展活力。进一步拓展开放的广度和深度，加快重要领域和关键环节的改革步伐。

　　提高对外开放水平。全方位扩大开放，招大引强，引进内外资金300亿元。大力引进项目、资金、技术、人才和先进管理经验。依托特色园区，主攻电子信息、战略性新兴产业和“6+1”产业招商，积极开展产业链招商、境外招商、配套基地招商。全面放开投资领域，在大力推进工业招商的同时，加大城市建设、农业、现代服务业等领域的招商力度，进一步提升开放型经济发展水平。完善招商工作机制，调动市直及县直单位参与招商引资、服务项目建设的积极性。发挥铁海联运、陆地港和出口加工区的作用，建立健全扩大出口的便捷通道。完善外贸出口激励政策，优化出口结构，积极发展加工贸易，保持外贸稳定增长。

　　深化重点领域改革。深化医药卫生体制改革，抓好县级公立医院综合改革试点，完成基层卫生医疗机构70%的债务化解任务，落实乡村医生各项补助政策，在每个村委会选择1所村卫生室实施国家基本药物制度。积极稳妥推进国有林场改革，进一步明晰国有林场产权，保障国有森林资源持续稳定增长。抓好国企改革后续工作，妥善解决遗留问题。抓好农村集体土地确权登记，明晰集体土地所有权和使用权。大力推进全民创业。加强政策引导，大力支持本地能人带头创业、外出人员返乡创业。鼓励城镇居民到农村建基地、搞开发，参与农业产业化经营。落实支持企业发展的优惠政策，加大对小微型企业的支持力度，鼓励和支持经营管理人员、科技人才、高校毕业生等各类人才投身创业。放宽市场准入，激发民间投资活力，支持民营资本进入市政公用事业、社会事业、金融服务等领域。

　　促进县域经济发展。鼓励和引导科技、人才、资金向县域经济发展倾斜，增强县域经济发展能力。积极探索促进县域经济发展的有效途径和政策措施，支持县市区发挥各自优势，发展壮大特色产业，形成块状经济。支持青原区、井冈山市、安福县分别抓好全省统筹城乡发展、县域经济发展、民营经济发展综合改革试点工作。完善对县市区的考评办法，注重可用财力和支出能力的考核，促进县市区在提高财政支出能力上进位赶超。

　　（七）加快基础设施建设，增强发展后劲。继续实施项目带动战略，保持投资对经济增长的强劲拉动。全年安排重点建设项目400个，计划完成投资550亿元。

　　大力推进项目建设。加强对重点建设项目的支持、协调和推进，加快一批项目开工建设和建成投产。道路交通方面，力争吉莲高速、抚吉高速、井睦高速建成通车，衡茶吉铁路主体工程完工，岳阳至吉安铁路、井冈山机场扩建等项目开工建设，昌吉赣铁路客运专线、吉安至厦门铁路、衡茶吉铁路东延、南昌至韶关高速公路等项目完成前期工作；105国道中心城区改道完成路基工程，永和连心大桥完成桥墩建设，青东公路改造二期主体工程完工；全市新建农村公路800公里。电力能源方面，力争赣江石虎塘航电枢纽主体工程竣工运行，峡江水利枢纽工程完成三期围堰截流，井冈山水电站实现一期截流，赣江新干航电枢纽开工建设；做好华能井冈山电厂三期、新干盐化城热电联产等项目的前期工作，争取遂川清秀山风电、泰和水槎风电获得核准并开工建设；实施农网改造升级工程，新建11个220千伏、110千伏输变电工程；推进省天然气管网二期工程建设，中心城区、吉安县、井开区、吉水县实现通气；续建中石化成品油管道二期工程，开工建设西气东输三线工程。公共服务设施方面，力争市博物馆与城市展示馆、市特殊教育学校竣工投入使用；市工人文化宫与妇女儿童活动中心、科技馆与青少年活动中心全面完工；市民服务中心、吉安新一中教学楼完成主体工程；吉州窑旧址公园开工建设；新白鹭洲中学基本完工；13个县市区卫生监督所竣工,３所县级医院综合大楼主体工程基本完工。

　　完善项目推进机制。以国家投资政策为导向，抓好重大项目包装、论证、申报工作，争取更多项目得到中央和省里支持。着力破解项目实施中的土地制约问题，大力推进造地增粮、土地增减挂工程以及有偿转让机制，争取更多建设用地指标。坚持领导挂重点项目制度、重点项目督查调度制度，保障项目高效有序推进。加强对重大项目建设资金的调度和前端预审及后端结算审计，保障资金需求，规范资金使用。严格项目建设程序，强化项目建设档案管理和全过程管理，确保工程建设质量与安全。

　　（八）以保障和改善民生为重点，加强和创新社会管理。坚持财力更多向民生倾斜，着力解决好群众最关心、最直接、最现实的利益问题，让广大人民群众更多享受改革发展成果。

　　切实保障和改善民生。认真办好省政府下达的70件民生实事。落实促进就业创业的各项政策，新增城镇就业5万人以上，新增农村劳动力转移就业7万人以上，发放小额担保贷款增加3亿元，工业园区定向培训3万人。扩大社会保险覆盖面，实现城乡居民养老保险全覆盖。建立健全职工工资、退休人员基本养老金、城乡低保标准的正常调整机制，城市低保、农村低保平均标准分别提高到350元和170元。继续实行儿童“两病”免费救治，全面实施困难尿毒症患者免费透析救治和重度聋儿实施人工耳蜗植入康复救治。推进“菜篮子”工程，保障主要农产品市场供应，改造提升中心城区菜市场和县市农贸市场。强化价格监测调控，严厉查处价格违法行为，确保物价基本稳定。

　　扎实推进保障性安居工程建设。有序出让土地，规范实施项目，抓好一批精品商住小区的开发建设，促进房地产业健康发展。实行经济适用住房、廉租房、公租房“三房合一、租售并举”，以公租房为重点，加大保障性安居工程建设力度，开工建设廉租房、公租房1.55万套，竣工1.32万套；改造农村困难群众危房1.04万户。完善保障性住房资格认定制度，让更多困难群众住房条件得到改善。

　　协调发展各项社会事业。加大教育投入，确保财政教育支出占当年公共财政支出比重达到16%。推进学前教育加快发展、义务教育均衡发展，开工建设6个县区22所中心幼儿园。大力发展职业教育，推进职业教育园区建设，开工建设吉安科技职业技术学院。以科技创新“六个一”工程为抓手，培育民营科技企业、高新技术企业，组建一批科技研发平台和优势团队。深入实施人才强市战略，加强专业技术人才和高技能人才队伍建设，对作出特殊贡献的人才实行津贴奖励制度。深入实施文化惠民工程和基层文化设施提升改造工程，扎实开展农村文化“三项活动”，精心组织创作一批优秀文艺精品。加快城乡社区体育设施建设，积极开展全民健身活动。完善城乡医疗卫生服务体系，推进新农合和大病统筹，提高城镇居民大病医疗保险最高支付限额。坚持不懈抓好人口与计划生育工作，实施优生促进工程，稳定低生育水平。扎实推进民兵预备役和国防后备力量建设，巩固军政军民团结。

　　加强污染治理和环境保护。严把项目建设环保准入关，对项目建设严格落实环保设施“三同时”制度。完善城镇污水收集管网，加快工业园区污水处理设施建设，新建遂川、万安、永丰、吉水、峡江等5个园区污水处理厂。推进农村环境综合整治，加强饮用水源地保护，控制农村面源污染。加快再生资源回收利用体系建设，促进资源循环利用。加大节能减排工作力度，全面推进工业、交通、建筑、公共机构和居民生活等领域的节能减排。

　　加强和创新社会管理。强化基层社会管理服务平台建设，推行社区网格化管理，构建以政府、社区、企业、社会团体和公民联动的社会管理机制。积极做好新形势的群众工作，健全维护群众权益机制。创新信访工作机制，建立市县乡村四级矛盾纠纷联动化解平台，妥善解决群众合理合法诉求。加大互联网监管力度，加强引导网络舆情。强化安全生产监管，重点抓好生产企业、各类学校、道路交通和高危行业的安全管理，严防重特大安全事故发生。开展质量兴市活动，创建全国食品药品安全示范区，提高质量和食品药品监管水平。加强应急管理工作，健全社会预警体系和应急机制，不断提高应对各类突发事件和保障公共安全的能力。强化社会治安综合治理，严密防范和依法打击各类违法犯罪活动，促进社会平安稳定。

　　各位代表，新形势下，人民群众对政府服务有更高期待，对政府工作有更高要求，加强政府自身建设和改革的任务更加繁重。我们一些领导干部和政府工作人员还没有牢固树立群众观点，为基层服务、为企业服务、为群众服务的意识还比较淡薄，解决具体问题的能力和办事效率还不够高；法制观念还需加强，从严执法的要求还需进一步落实，依法行政的能力和水平亟待提高；有些部门不同程度存在治政不严、管理不力、懒散松懈的问题，工作中还存在被动应付、得过且过、扯皮推诿、行政不作为等现象，极少数政府工作人员甚至以权谋私、贪污腐败。我们一定认真负责地对待这些问题，不断深化“思想大解放、作风大转变、服务大提升”主题教育活动，尽最大努力切实解决这些问题。坚持以人为本，努力建设情系百姓的为民政府。强化干部的亲民、爱民、为民意识，从群众最期盼、反映最强烈的问题抓起，尽心竭力为百姓办好事、办实事。推进经济发展更多地关注民生民计，加快城市建设更多地考虑利民为民，强化社会管理更多地体现人文关怀。推进依法行政，自觉接受人大及其常委会的法律监督、工作监督和政协的民主监督，主动接受社会监督和舆论监督。坚持科学民主决策，严格落实公众参与、专家咨询论证、社会风险评估等制度，加强对重大决策的跟踪督查。对事关经济社会发展全局和与群众切身利益密切相关的重大问题，实行社会公示和听政制度，推进政务信息公开和新闻发布，做到议政于民。畅通民情通道，倾听群众诉求，切实解决群众的实际问题。坚持提升效能，努力建设勤政快捷的高效政府。加强部门协作和效能问责，强化维护发展环境的责任意识。倡导精细、务实的工作作风，以机关效能建设为抓手，集中整治影响发展环境的突出问题，切实纠正“庸懒散、假浮蛮、私奢贪”等不良作风。强化深入实际攻坚克难的进取意识，深入矛盾集中的一线调查研究，更多地现场办公解决问题、推动工作。进一步创新行政服务方式，规范市、县行政服务中心和乡镇便民中心建设，更好地方便群众办事。认真落实重大项目绿色通道服务制度，推行重大产业项目“全程式”服务，提升项目服务水平。进一步深化行政审批制度改革，行政审批事项精简30%，全面推进网上审批、电子监察运行系统，提高办事效率。坚持从严治政，努力建设风清气正的廉洁政府。认真落实政府部门党风廉政建设责任制，规范岗位廉能管理，形成风险岗位廉能管理长效机制。加快重点领域预防腐败制度建设，深化工程建设、土地出让、房地产开发等领域突出问题的专项治理，加大行政监察和审计监督力度，严肃查处违法违纪行为，坚决纠正损害群众利益的不正之风。坚持艰苦奋斗、厉行节约，将有限财力更多投向民生社会事业。

　　各位代表！做好今年各项工作，任务艰巨，责任重大。让我们紧密团结在以胡锦涛同志为总书记的党中央周围，在省委、省政府和市委的正确领导下，大力弘扬井冈山精神，齐心协力，扎实苦干，为建设开放繁荣秀美幸福新吉安作出新的更大贡献，以优异成绩迎接党的十八大胜利召开！
